# Posterior Circulation ASPECTS on CT Angiography Predicts Futile Recanalization of Endovascular Thrombectomy for Acute Basilar Artery Occlusion

**DOI:** 10.3389/fneur.2022.831386

**Published:** 2022-03-16

**Authors:** Keni Ouyang, Zhiming Kang, Zhengxing Liu, Botong Hou, Jiabing Fang, Yu Xie, Yumin Liu

**Affiliations:** Department of Neurology, Zhongnan Hospital, Wuhan University, Wuhan, China

**Keywords:** basilar artery occlusive, endovascular thrombectomy, futile recanalization, CT angiography, posterior circulation Acute Stroke Prognosis Early computed tomography score, onset-to-puncture time

## Abstract

**Background:**

Acute basilar artery occlusion (BAO) is the most potentially disastrous outcome and has a high risk of recurrence stroke in posterior circulation infarction (PCI). However, the rate of futile recanalization remains high despite successful recanalization. The objective of this study was to investigate 90 days functional outcomes among patients with BAO who underwent endovascular thrombectomy (EVT) and to identify the risk factors associated with futile recanalization.

**Methods:**

We retrospectively analyzed 72 patients with acute BAO who received EVT from January 2018 to June 2021. CT angiography source images posterior circulation Acute Stroke Prognosis Early CT Score (CTA-SI pc-ASPECTS) evaluated the extensive hypoattenuation in patients with BAO. Futile recanalization defined an modified Rankin Scale (mRS) of 3-6 at 90 days despite a successful recanalization. Logistic regression analysis was performed to investigate the predictors of futile recanalization.

**Results:**

Our sample included a total of 55 eligible patients. Patients with poor outcomes showed that the pc-ASPECTS score was lower in patients with poor outcomes than that in patients with good outcomes (*P* = 0.017). Longer time from symptoms onset-to-the puncture (*P* = 0.014) and elevation of leucocytes (*P* = 0.012) were associated with poor outcomes. The multivariable logistic analysis showed that pc-ASPECTS and onset-to-puncture time (OPT) were independent predictors of futile recanalization.

**Conclusions:**

This study suggested that pc-ASPECTS and OPT are independent predictors of futile recanalization after EVT in patients with BAO. The lower pc-ASPECTS score and longer puncture time will have a poor clinical outcome.

## Introduction

Posterior circulation infarction (PCI) accounts for 20–25% of all incidents of acute ischemic stroke (AIS) (1) and has complex clinical presentations. Acute basilar artery occlusive (BAO) is the most devastating form of PCI (2), with potentially disastrous outcomes and a high risk of recurrent stroke (3). The mortality of patients with AIS caused by BAO is more than 85%. In the past few decades, the availability and technical improvement of endovascular thrombectomy (EVT) have changed this devastating scenario (4). BAO has developed from an almost fatal disease to become a treatable disease. However, there is a phenomenon that despite a successful recanalization, the outcome is poor; we define it as futile recanalization. The recent completed ENDOSTROKE study showed that only 34% of patients with BAO achieved good clinical outcomes despite a 79% rate of recanalization with EVT (5). Besides this, the results of a recent study suggested that this phenomenon occurred more often in BAO than in anterior circulation large-vessel occlusion (LVO) (6). Although the mechanism of futile recanalization has not yet been clarified, it might be associated with age, stroke severity, time from stroke onset to treatment, infarct distribution and volume, brain edema, leukoaraiosis, collateral status, subacute re-occlusion, microvascular compromise, impaired cerebral autoregulation, etc (7). In addition, pre-treatment imaging changes, both on MRI and CT, were indicated as independent predictors of clinical outcomes in recent studies (5, 8–10), highlighting the importance of applying initial prognostic markers for patient selection to avoid futile recanalization (4). Therefore, it is very important to select appropriate patients with BAO to receive EVT to avoid futile recanalization.

Recent studies found that the appropriate selection of patients for EVT upon imaging criteria helps improve prognosis (7). Areas of hypoattenuation on CT angiography (CTA) delineate regions of brain tissue with ischemic damage (11, 12). Compared with non-contrast CT, CTA source images (CTA-SI) is more accurate in predicting the final extent of infarction and clinical outcomes (8, 11, 12). The posterior circulation Acute Stroke Prognosis Early CT Score (pc-ASPECTS), first proposed by Puetz et al. (13), is a semi-quantitative method to grade irreversible ischemia in the vertebrobasilar artery system (14). Several studies that have applied pc-ASPECTS to CTA-SI have validated its usefulness and demonstrated that it could predict functional outcomes in patients with AIS caused by BAO (12, 13, 15). Previous studies have emphasized the importance of pc-ASPECTS on the outcomes of patients with BAO after EVT; a high pc-ASPECTS (pc-ASPECTS ≥8) on the initial image usually implies a higher quality of life and lower mortality rates. Nevertheless, recent data demonstrated that a large number of patients with scores below this threshold may still recover well after EVT if recanalization occurs rapidly.

In this study, we aimed to assess the 90-day functional outcomes among patients with BAO who underwent EVT and to identify the risk factors associated with futile recanalization, focused on pc-ASPECTS and OPT, for screening out patients who cannot benefit significantly from EVT.

## Methods

### Patients

We retrospectively analyzed 72 consecutive patients with acute BAO who received EVT from January 2018 to June 2021 in Zhongnan Hospital of Wuhan University.

The inclusion criteria included: (1) patients were confirmed of BAO by CTA; (2) ≥18 years old; (3) onset within 24 h; (4) successful recanalization after EVT [modified Thrombolysis in Cerebral Infarction (mTICI) ≥2b]; (5) complete 90-day follow-up was available; (6) all signed informed consent for treatment.

**Figure 1:**
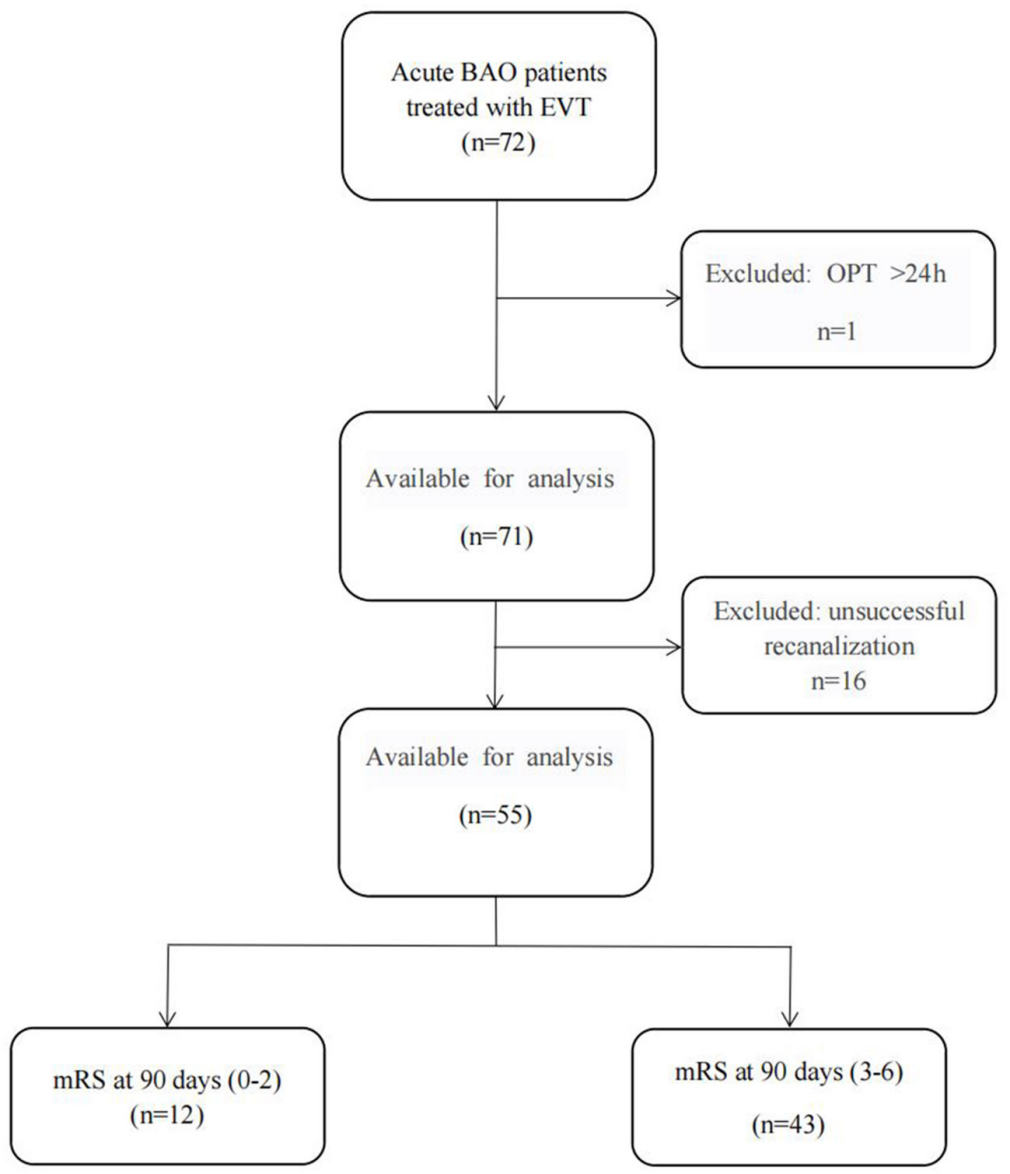
The flow chart for the inclusion of patients. (BAO, basilar artery occlusive; EVT, endovascular thrombectomy; OPT, Onset-to-Puncture Time; mRS, the modified Rankin scale).

The exclusion criteria included: (1) pre-stroke modified Rankin Scale (mRS) > 3; (2) patients with anterior circulation stroke; (3) CTA were a known history of contrast medium allergy or any degree of renal failure; (4) intracranial hemorrhage or tumor. The flow chart for the inclusion of patients is summarized in Figure 1.

This study was carried out in compliance with the Declaration of Helsinki (10) and was approved by the ethics committees of Zhongnan Hospital of Wuhan University. All of the information was anonymous and confidentiality of information was assured (10).

### Clinical Data Collection

Including demographic data, the severity of symptoms [National Institute of Health Stroke Scale (NIHSS)], stroke risk factors (hypertension, diabetes mellitus, previous stroke, smoke, hyperlipidemia, and atrial fibrillation), OPT, surgical methods, mTICI, [successful recanalization was defined as final mTICI of 2b to 3 (14)], hemorrhagic transformation [small or confluent petechiae, with or without a space-occupying effect in the infarcted area with or without clinical deterioration (16)], and mRS at 90 days [functional outcome at 90 days were derived from telephone or outpatient follow-up with patients or their relatives (13)].

Ischemic events were identified by two experienced neurologists based on the medical history description and brain CT or MRI (10). Clinical evaluations were conducted by three professional investigators, without knowledge of the baseline performance and surgical details, through clinical interviews or standardized telephone interviews with patients or their relatives (17). mRS was used to record the EVT treatment of the patient's clinical state. A favorable outcome was defined as an mRS score of 0–2, a poor outcome was defined as 3–6 (17).

### Imaging Analysis

**Figure 2:**
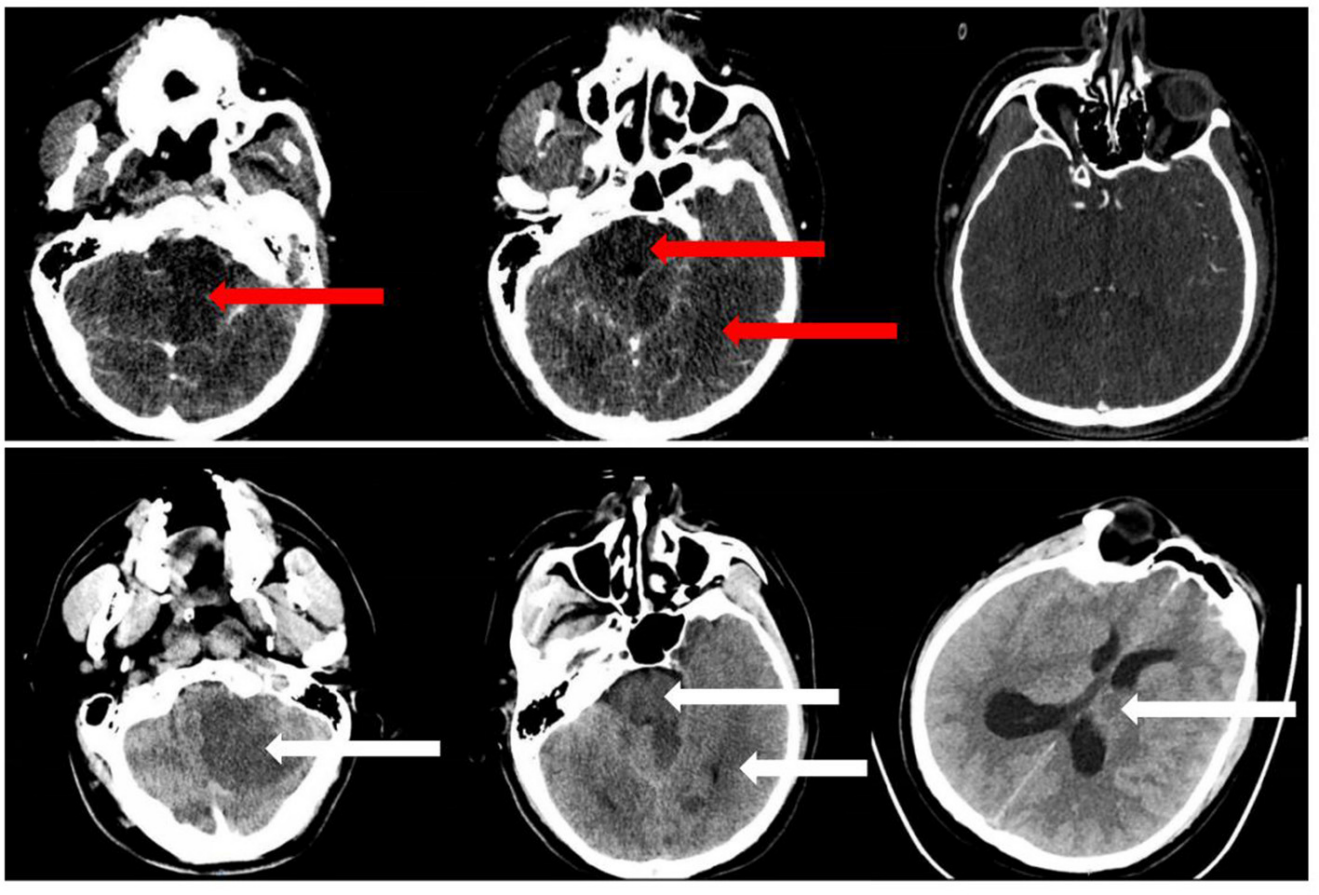
Example of a patient with BAO in our study. We rated CT angiography (CTA) source images (CTA-SI) (upper row) posterior circulation Acute Stroke Prognosis Early CT Score (pc-ASPECTS) score 6 (hypoattenuation left PCA territory, left cerebellum, midbrain) and follow-up non-contrast CT (NCCT) (lower row) pc-ASPECTS score 5 (additional ischemic change left thalamus).

Posterior circulation Acute Stroke Prognosis Early CT Score (Pc-ASPECTS) allots the posterior circulation with 10 points (13). Pc-ASPECTS was scored early ischemic changes by evaluating hypoattenuation areas in posterior circulation territories on CTA-SI. One point each is subtracted for early ischemic changes in the left or right thalamus, cerebellum, or posterior cerebral artery (PCA) territory, respectively, and two points in any part of the midbrain or pons (18) (Figure 2). A pc-ASPECTS score of 10 indicates the absence of visible posterior circulation ischemia, a score of 0 indicates hypoattenuation in all pc-ASPECTS territories.

The imaging data were evaluated by two experienced neuro-radiologists without knowing the clinical data in advance. In cases of disagreement, a higher-level neuro-radiologist will review and make the final judgment. We rated the perfusion of the entire basilar artery (BA) with a delayed flow or full perfusion with a normal BA flow as described in the digital subtraction Angiography (DSA) results (according to mTICI 2b to 3 flow grades) as BA recanalization (19).

### Clinical Outcomes

The mRS was used to assess the 90-day functional outcomes (20). Without knowing the patient's clinical information, a follow-up was conducted by professional medical researchers according to developed interview protocols. The primary outcome was futile recanalization after EVT defined as an mRS of 3–6 at 90 days despite successful recanalization.

### Statistical Analysis

Data were collected on standard forms, evaluated for completeness (21). Continuous variables were presented as mean ± SD or median with interquartile range (IQR). Categorical variables were presented as counts and percentages. In the univariate analysis, the continuous variable is abnormally distributed, the Mann-Whitney *U*-test or student *t*-test was used to determine differences between the two groups. The differences between the two groups of categorical variables were tested by χ^2^ test or Fisher's exact test. For variables with *P* < .1 in the univariate analysis, multivariate logistic regression with a forward stepwise method was performed. The receiver operating characteristic (ROC) test was used to evaluate the predicted area under the curve (AUC) and identify the best cutoff value to distinguish between poor and favorable clinical outcomes.

A two-sided *P*-value of .05 or less was considered statistically significant. All statistical analyses were performed using IBM SPSS Statistics 26 software (Version 26.0; IBM) (17).

## Results

**Figure 3:**
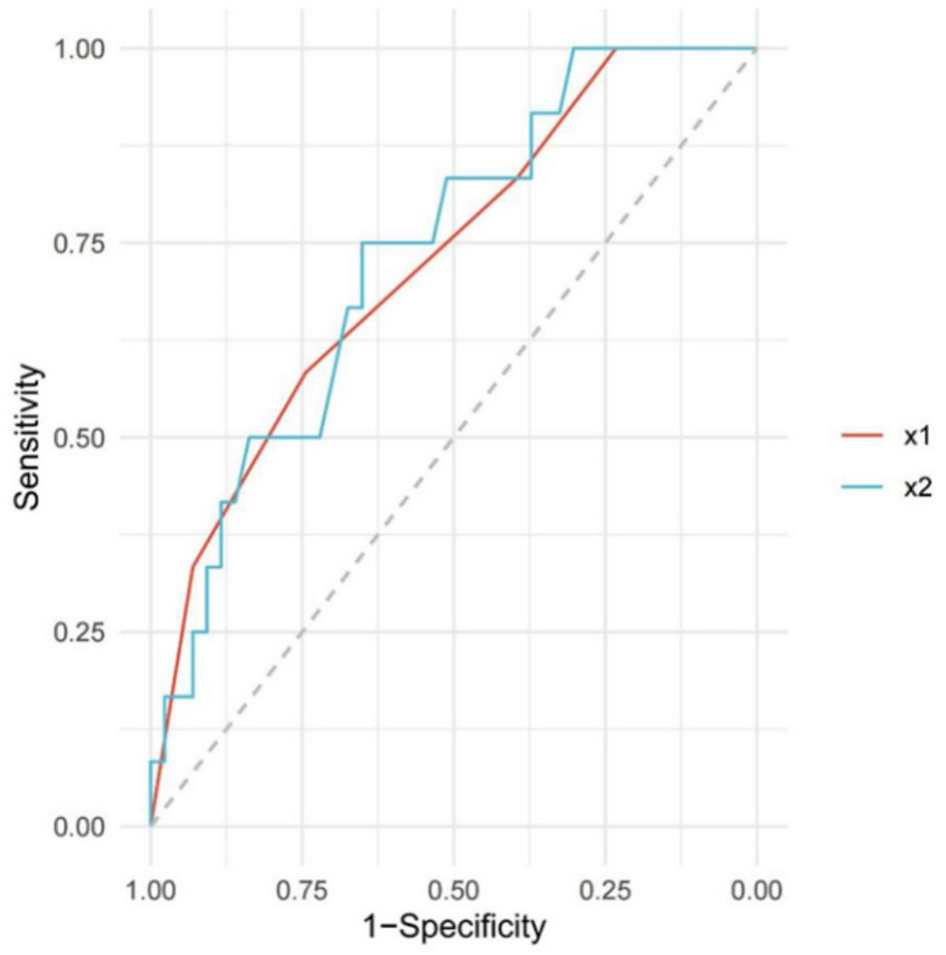
Receiving operating characteristic (ROC) curve of pc-ASPECTS and OPT. The red solid line (x1) represents the boundary of the pc-ASPECTS area under the curve (AUC), and the blue band (x2) represents the boundary of onset-to-puncture time (OPT). The predictive performance indexes of this scale at different cutoff values are shown in Table 3. ROC, Receiver-operating Characteristic; OPT, Onset-to-puncture time; AUC, Authentication Center.

**Figure 4:**
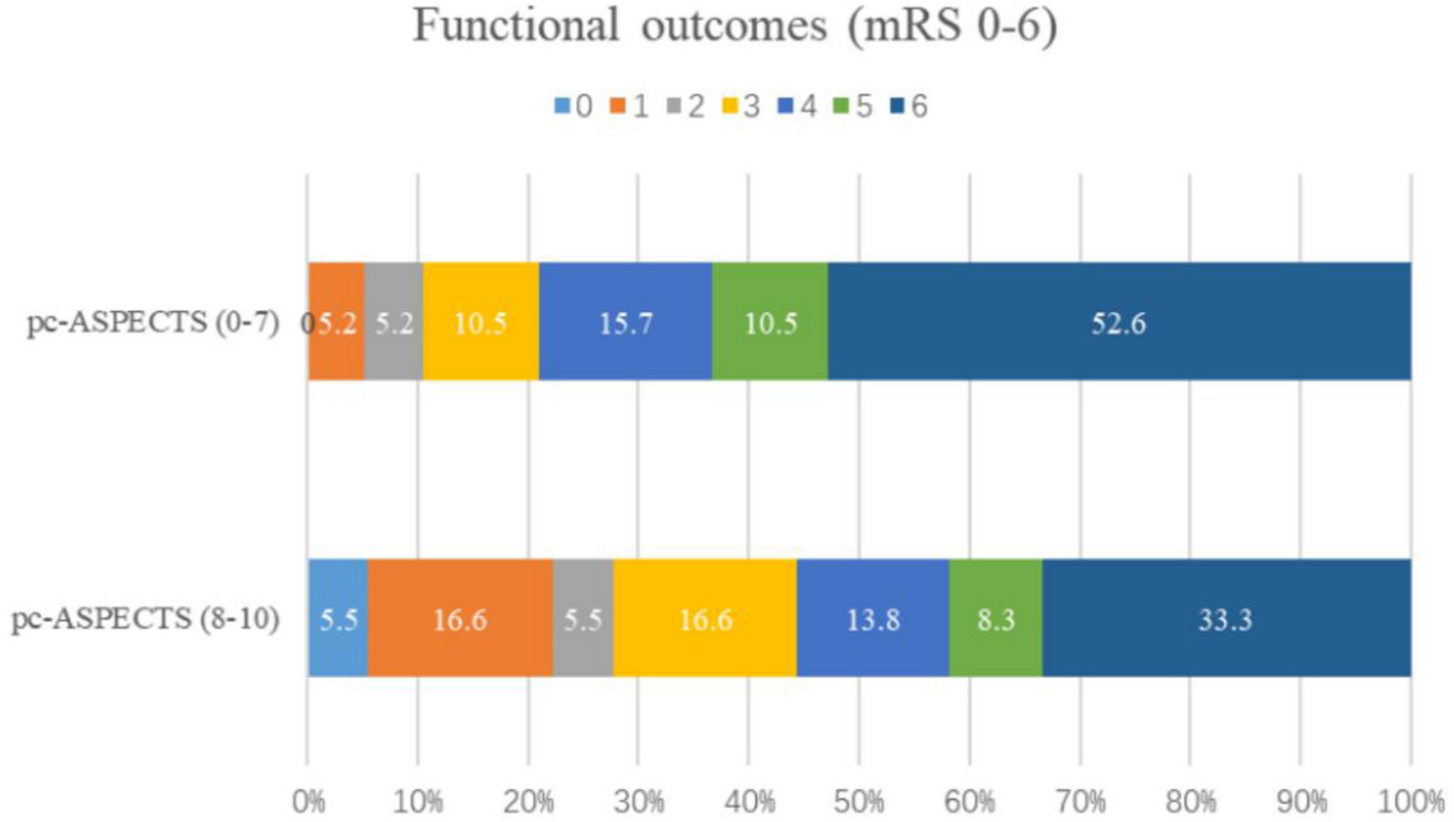
The distribution of the modified Rankin scale (mRS) at 90 days in patients with BAO in two groups. Numbers in bars indicate the percentage of patients. mRS, modified Rankin Scale; pc-ASPECTS, the posterior circulation Acute Stroke Prognosis Early CT Score.

The baseline characteristics of the patients are presented in Table 1. A total of 55 patients who fulfilled the inclusion criteria were included. The average age of the patients was 64 years (ranging from 39 to 90), including 47 males (85.4%). The pretreatment NIHSS score for all patients was 35 (IQR, 20–35). The median pc-ASPECTS was 8 (IQR, 7–9), and the time from stroke onset-to-puncture was 480 min (IQR, 345–700). Among them, 12/55 patients (21.8%) were classified into the favorable outcome and 43/55 patients (78.2%) had a poor outcome. Comparison of predictors of clinical outcomes by univariate analysis (favorable vs. poor). The pc-ASPECTS scores in patients with poor outcomes was lower than those of patients with good outcomes [8 (7–9) vs. 9 (8–10); *P* = 0.017]. The time from symptoms onset to the puncture [510 (410–770) vs. 383 (227–490); *P* = 0.014] and leukocyte (10.1 (7.6–12.2) vs. 7.0 (6.3-9.1); *P* =.012) were associated with clinical outcomes. The predictors of the functional outcome by multiple logistic regression analysis were listed in Table 2. The pc-ASPECTS (OR,.476; 95% CI,.227-.998, *P* =.049) and OPT (OR, 1.004; 95% CI, 1.000–1.007, *P* =.048) were independent predictors for functional outcome. As a result of ROC curve analysis (22), the AUC for the pc-ASPECTS was.726 (95% CI,.566–0.885) and for the time from onset-to-puncture was.734 (95% CI,.582–0.885). The best cutoff value respectively was 8 score and 476 min, to maximize the sensitivity and specificity for discriminating patients with good outcomes and poor outcomes (22) (Figure 3, Table 3). The distribution of 90-day-mRS according to the categorized pc-ASPECTS score are presented in Figure 4. We can see that patients with lower pc-ASPECTS (0–7) accounts for a larger proportion of poor outcome.

**Table 1:** Characteristics and clinical data of the BAO patients treated with EVT.

**Variables**	**All patients (*n* = 55)**	**Favorable outcome (*n* = 12)**	**Poor outcome (*n* = 43)**	***P*** **-value**
Male, *n* (%)	47 (85.4%)	9 (75.0%)	38 (88.4%)	0.485
Age, y (median, IQR)	64 (57–75)	69 (63–75)	62 (57–75)	0.103
Hypertension, *n* (%)	38 (69.1%)	8 (66.7%)	30 (69.8%)	1.000
Diabetes, *n* (%)	14 (25.4%)	3 (25.0%)	11 (25.6%)	1.000
Prior stroke, *n* (%)	11 (20.0%)	4 (33.3%)	7 (16.3%)	0.369
Smoking, *n* (%)	18 (32.7%)	4 (33.3%)	14 (32.6%)	1.000
Hyperlipidemia, *n* (%)	6 (10.9%)	3 (25.0%)	3 (25.0%)	0.212
Atrial fibrillation, *n* (%)	8 (14.5%)	2 (16.7%)	6 (14.0%)	1.000
Coronary heart disease, *n* (%)	9 (16.4%)	4 (33.3%)	5 (11.6%)	0.175
pc-ASPECTS, (median, IQR)	8.0 (7.0–9.0)	9.0 (8.0–10.0)	8.0 (7.0–9.0)	0.017^*^
NIHSS score, (median, IQR)	35.0 (20.0–35.0)	34.5 (18.0–36.0)	35.0 (20.0–35.0)	0.532
Blood glucose, mmol/L (median, IQR)	8.6 (7.0–10.2)	8.3 (6.7–9.4)	8.8 (7.0–10.7)	0.354
Leukocyte, 10^9^/L (median, IQR)	9.5 (7.2–11.8)	7.0 (6.3–9.1)	10.1 (7.6–12.2)	0.012^*^
Intravenous r-tPA, *n* (%)	17 (30.9%)	5 (41.7%)	12 (27.9%)	0.576
Onset to puncture, min (median, IQR)	480 (345–700)	383 (227–490)	510 (410–770)	0.014^*^
Onset to recanalization, min (median, IQR)	594 (475–867)	551 (319–686)	604 (524–907)	0.053^*^
Puncture to recanalization, min (median, IQR)	104 (75–151)	90 (66–219)	110 (75–141)	0.610
mTICI, 2b or 3, *n* (%)				0.162
2b	21 (38.2%)	2 (16.7%)	19 (44.2%)	
3	34 (61.8%)	10 (83.3%)	24 (55.8%)	
Type of procedure, *n* (%)				0.276
Aspiration alone	14 (25.5%)	5 (41.7%)	9 (20.9%)	
Stent retrieve	17 (30.9%)	2 (16.7%)	15 (34.9%)	
Angioplasty	24 (43.6%)	5 (41.7%)	19 (44.2%)	
Hemorrhagic transformation, *n* (%)	8 (14.5%)	2 (16.7%)	6 (14.0%)	1.000

* *Variables with p-value < 0.1*.

*BAO, basilar artery occlusive; EVT, endovascular thrombectomy; pc-ASPECTS, the posterior circulation Acute Stroke Prognosis Early CT Score; NIHSS, National Institutes of Health Stroke Scale; mTICI, modified Thrombolysis in Cerebral Infarction Score; IQR, interquartile range*.

**Table 2:** Independent risk factors for poor outcome at 90 days.

**Variables**	**β**	**Odds Ratio**	**Standard Error**	**95% CI**	***P*** **-value**
pc-ASPECTS, median (IQR)	−0.743	0.476	0.378	0.227–0.998	0.049^*^
OPT, median (IQR), min	0.004	1.004	0.002	1.000–1.007	0.048^*^
Leukocyte, median (IQR), × 10^9^/L	0.351	1.421	0.180	0.998–2.023	0.051

* *Variables with p-value < 0.05*.

*IQR, interquartile range; OPT, onset to puncture time; β, regression coefficient; 95% CI, 95% Confidence Interval*.

**Table 3:** ROC curve analysis of pc-ASPECTS and OPT.

**Variables**	**AUC value**	**95% CI**	**Best Cutoff Value**	**Sensitivity**	**Specificity**
pc-ASPECTS, median (IQR)	0.726	0.566–0.885	8	0.583	0.744
OPT, min, median (IQR)	0.734	0.582–0.885	476	0.651	0.75

*AUC indicates area under the curve; mRS, modified Rankin Scale; pc-ASPECTS, the posterior circulation Acute Stroke Prognosis Early CT Score; OPT, onset to puncture time; IQR, interquartile range; 95% CI, 95% Confidence Interval*.

## Discussion

Severe ischemic cerebrovascular diseases (ICD) not only bring physical diseases but also life pressure to patients and their families. BAO accounts for about 1% of all strokes (22) and for 5% of all intracranial large vessel occlusions (LVO) (18). Although the efficacy of EVT for BAO has been demonstrated in multiple observational studies (6, 9, 14, 23), its positive impact on the BA is still a matter of debate (23). Even if successful recanalization is strongly associated with a high mortality rate and a high risk of disability, these treatments are often expensive, with a poor outcome. Reliable predictions of futile recanalization are important to identify patients with BAO who do not benefit significantly from EVT and assist in the personalized formulation.

The main 2 findings of the study were as follows. (1) the onset-to-puncture time and pc-ASPECTS on CTA-SI before treatment were independent risk factors in predicting futile recanalization for patients with BAO who underwent EVT. (2) The best cutoff value for differentiation between favorable outcomes and poor clinical outcomes was a score of 8 for pc-ASPECTS and 476 min (≈8 h) for the time from onset-to-puncture before treatment.

The recently published studies found that the appropriate selection of patients for EVT upon imaging criteria helps improve prognosis (2, 4, 12, 16). In this retrospective study, our study demonstrates that pc-ASPECTS on CTA-SI can help predict the functional outcomes 15 after BAO successful recanalization (OR, 0.476; 95% CI, 0.227–0.998, *P* = 0.049) and being dichotomized at ≥8 vs. <8 to predict favorable outcome and poor outcome at 90 days. Patients with lower scores often represented extensive brain ischemic infarction lesions, suggesting that despite successful recanalization, it is difficult to achieve good functional outcomes.

It has shown that the quantification of hypoattenuation on CTA-SI predicts clinical outcomes in patients with posterior circulation stroke (PCS) (12). Using a systematic approach with a novel CT score (12, 13), pc-ASPECTS is a semi-quantitative grading system for PCS suggested by Puetz et al. in 2008 (13). It is relatively simple and easy to apply (24). Based on previous findings indicating that the numbers of regions involved and the involvement of the pons and midbrain are the most critical issues in functional outcome in patients with PCS (25). Applied on CTA-SI, pc-ASPECTS predicted clinical outcomes in patients with suspected BAO. Patients with extensive hypoattenuation defined by a CTA-SI pc-ASPECTS score <8 usually represent futile recanalization (7). In our study, patients with lower pc-ASPECTS (0–7) accounts for a larger proportion of poor outcome, the overall functional outcome was comparable to the average outcome reported in the literature (26, 27).

Basilar artery (BA) recanalization, thrombus location, length of BA obstruction, and state of collaterals have been identified as independent variables affecting functional outcomes in several studies (28, 29). However, no criteria are currently available to identify patients who will likely benefit from EVT. MRI with diffusion-weighted imaging (DWI) sequences is considered the diagnostic “Gold standard” in patients with PCS (30). However, the feasibility of MRI may be limited in these frequently unstable patients (29), and it takes a long time and costs a lot. A completely normal non-contrast CT (NCCT) scan seems ideal in terms of potential benefit (because no damage at that time) from any possible therapies but may introduce diagnostic uncertainty for the stroke neurologist (12). The comparison of CTA, CTP, and multimodal MRI to predict outcomes and treatment response in patients with BAO could be the subject of future studies (13, 31) and more research is needed to investigate the most reliable scoring system.

Another key finding was the strong effect of time to treatment on poor clinical outcomes. There is an old saying that goes that time is money and in our research, we were surprised to find that time is also life. The time to treatment initiation is generally held as the single most dominant determinant of the fate of ischemic brain tissues (32); some centers with smaller cohorts of BAO have found an association between OPT and outcomes (28). Furthermore, Mokin et al. (33) reported that the 6 h window cutoff might be an important criterion for the prediction of outcomes in patients with PCS treated with EVT (32). But, in our analysis, the 476 min (8 h) window cutoff from symptom onset-to-puncture was statistically significant. The longer the puncture time, the more likely to have a poor outcome (OR, 1.004; 95% CI, 1.000–1.007; *P* = 0.048). This result first suggests that each patient may have a unique best treatment time window and we should make the right decision as soon as possible to reduce transport and preoperative preparation time. Secondly, due to the misleading nature of prodromal symptoms related to BAO, clinical diagnosis is often delayed, leading to prolonged time intervals to imaging evaluation and treatment (18). Finally, currently known puncture methods include radial artery and femoral artery puncture. The radial artery is small and difficult to find, which is more difficult for inexperienced doctors. Therefore, whether femoral artery puncture is more rapid and safe needs further study. In fact, our findings were markedly different from the BEST trial, which failed to show benefit of EVT within 8h. In the BEST registry, patients typically had higher clinical severity (34), which increased the chance of selection bias, and the study that had been completed 10 years ago was not applicable to contemporary clinical. Moreover, the results may have been confused by a loss of balance during the study.

Evidence showed that pc-ASPECTS was a more critical factor influencing the benefits from EVT over time (35). This is probably due to the patients who survived several hours after symptom onset, there may be a robust collateral arterial network maintaining brittle patency of brain stem perforators, which may improve treatment benefits from delayed recanalization (36, 37). However, each patient likely has a unique optimal time window for therapy because of inherent variability in collateral support (14). Hence, it is necessary to receive treatment as soon as possible. Moreover, patients with lower pc-ASPECTS often had a much longer time from onset-to-puncture, as indicated by our results, providing evidence that the chance of a favorable pc-ASPECTS (≥8) will probably decrease with time delay, resulting in deleterious effects (14). We have reason to assume that the longer the time spent, the larger the ischemic area is. If the OPT occurs rapidly, even patients with extensive cerebral ischemia and below the best cutoff point can still benefit from EVT. Compared to the previous reports (34, 38), the BEST trial and BASICS trial showed that there were no significant differences in the acute BAO outcome. Imaging selection was not clearly mentioned in the two trials that might be the cause of the negative outcomes, and the two trials recruitment programs were largely flawed, resulting in a large selection bias that could offset the potential benefits of EVT.

However, the effectiveness of pc-ASPECTS in its application on patients with acute BAO selection remains controversial, although it is might be the most widely used scale for PCS. Several DWI scoring systems have been published for the assessment of early ischemic injury in patients with BAO (13, 39–41). The Pons-Midbrain and Thalamus (PMT) score was published recently. It was a DWI-based semi-quantitative scale in which the infarctions of pons, midbrain, and thalamus were fully considered in assessing the outcome of EVT in patients with BAO (39). Future studies should reinforce assessing whether multimodal scores provide superior prognostic information in patients.

The major limitations of this study are the single-center retrospective design and that the clinical outcome was identified retrospectively and requires prospective validation in another cohort. Secondly, the lack of data regarding the etiology of the occlusion (embolism vs. thrombotic), thrombus volume, and collateral circulation (32). Finally, the relatively small sample size and selection bias (2).

## Conclusions

The results of this study indicate that pc-ASPECTS and OPT are independent predictors of futile recanalization after EVT in patients with BAO. We describe pc-ASPECTS which quantifies early ischemic changes in the posterior circulation (13). Applied on CTA-SI, pc-ASPECTS predicts futile recanalization after EVT and identifies patients with BAO who potentially benefit from EVT. The effect of OPT on EVT outcome is also described in our study; extending OPT not only increases the incidence of futile recanalization but also decreases functional independence. We consider that the chance of favorable pc-ASPECTS will probably decrease with OPT delay, resulting in detrimental effects.

## Data Availability Statement

The raw data supporting the conclusions of this article will be made available by the authors, without undue reservation.

## Ethics Statement

Ethical review and approval was not required for the study on human participants in accordance with the local legislation and institutional requirements. The patients/participants provided their written informed consent to participate in this study.

## Author Contributions

KO and ZK contributed to the conception and design of the study. YX revised some of the manuscript. YL conducted statistical analysis. KO, ZK, and JF organized the database. All authors participated in revising the article, reading it, and approving the submitted version.

## Funding

This work was supported by the Translational Medicine and Interdisciplinary Research Joint Fund of Zhongnan Hospital of Wuhan University (Grant Number: ZNLH201909).

## Conflict of Interest

The authors declare that the research was conducted in the absence of any commercial or financial relationships that could be construed as a potential conflict of interest.

## Publisher's Note

All claims expressed in this article are solely those of the authors and do not necessarily represent those of their affiliated organizations, or those of the publisher, the editors and the reviewers. Any product that may be evaluated in this article, or claim that may be made by its manufacturer, is not guaranteed or endorsed by the publisher.
